# Insidious Insights: Implications of viral vector engineering for pathogen enhancement

**DOI:** 10.1038/s41434-021-00312-3

**Published:** 2022-03-10

**Authors:** Jonas B. Sandbrink, Ethan C. Alley, Matthew C. Watson, Gregory D. Koblentz, Kevin M. Esvelt

**Affiliations:** 1 Nuffield Department of Medicine, University of Oxford, Oxford, UK; 2 Future of Humanity Institute, University of Oxford, Oxford, UK; 3 Media Laboratory, Massachusetts Institute of Technology, Cambridge, MA USA; 4 Center for Health Security, Johns Hopkins Bloomberg School of Public Health, Baltimore, MD USA; 5 Schar School of Policy and Government, George Mason University, Fairfax, VA USA

**Keywords:** Gene therapy, Virology, Genetic vectors, Genetic engineering

## Abstract

Optimizing viral vectors and their properties will be important for improving the effectiveness and safety of clinical gene therapy. However, such research may generate dual-use insights relevant to the enhancement of pandemic pathogens. In particular, reliable and generalizable methods of immune evasion could increase viral fitness sufficient to cause a new pandemic. High potential for misuse is associated with (1) the development of universal genetic elements for immune modulation, (2) specific insights on capsid engineering for antibody evasion applicable to viruses with pandemic potential, and (3) the development of computational methods to inform capsid engineering. These risks may be mitigated by prioritizing non-viral delivery systems, pharmacological immune modulation methods, non-genetic vector surface modifications, and engineering methods specific to AAV and other viruses incapable of unassisted human-to-human transmission. We recommend that computational vector engineering and the publication of associated code and data be limited to AAV until a technical solution for preventing malicious access to viral engineering tools has been established.

## Introduction

In vivo gene therapy holds great promise for treating many genetic disorders. Several gene therapy products have been licensed to date, allowing the treatment of previously incurable genetic diseases [1]. Many of these rely on viral vectors, modified non-pathogenic viruses used to deliver the encoded gene of interest to target cells. Further optimization of these delivery vehicles promises to improve their effectiveness and target further diseases.

However, certain biotechnologies exhibit “dual-use” potential, as they may inform and enable pathogen engineering by malicious actors [2]. Managing and mitigating such dual-use risks from biotechnologies is not only important to protect human health and society from global biological threats, but also to safeguard future technological advances from mistrust and poorly-planned restrictions.

While directly enhancing the virulence of a known pathogen poses the most obvious potential for misuse, viral vector research may generate insights relevant to pathogen engineering that contribute to lowering the barrier for viral engineering and synthesis. In a world where many viruses are constrained by pre-existing immunity or suboptimal adaptation to human receptors, genetically encoded methods of immune modulation or targeted tissue tropism could plausibly transform an innocuous virus into the cause of a new pandemic. Indeed, efforts to engineer viral immune evasion were part of at least one historical biological weapons program [3]. Insights applicable to viruses capable of autonomous human-to-human transmission are particularly concerning as they could plausibly generate transmissible agents posing global pandemic threats. In contrast, the direct misapplication of non-transmissible, non-replicating viral vectors for delivery of harmful payloads - the risk of which has been discussed previously [4, 5] - could harm individuals but would be unlikely to cause a global catastrophe.

Here, we highlight areas of viral vector research that may pose risks of enhancing or generating novel pandemic pathogens, including (1) universal genetic elements for immune modulation, (2) capsid engineering for antibody evasion, and (3) general-purpose viral engineering methodologies.

## Risks from the development of universal genetic elements for immune modulation

To evade immune responses to vectors and transgene products, researchers are exploring mechanisms to genetically engineer viral vectors to modulate the induction of host immune responses through the insertion of distinct genetic elements. For instance, to prevent detection of viral nucleic acids by intracellular sensors, Chan et al. inserted short non-coding DNA sequences into the AAV genome to directly antagonize the activation of the TLR9 immune sensor and “cloak” the much larger AAV DNA sequence from detection [6]. Similarly, the insertion of the US6 glycoprotein from CMV [7] and infected cell protein 47 (ICP47) from HSV [8], which inhibit the MHC class I pathway and prevent cell surface presentation of viral peptides for CTL targeting, have been investigated for immune modulation of AAV [9]. These and similar genetically encodable immune modulation approaches may be transferable to any virus exhibiting sufficient genomic flexibility: they involve the insertion of distinct genetic elements which function independently from existing viral machinery. Notably, the application of such generalizable approaches for immune modulation to potential pandemic pathogens requires little additional work and relevant expertise. While current immunoevasive approaches are still limited in efficacy and generalisability, efforts to improve their effectiveness are deeply concerning in light of society’s demonstrated vulnerability to pandemic viruses.

## Risks from capsid and envelope glycoprotein engineering

**Fig. 1:**
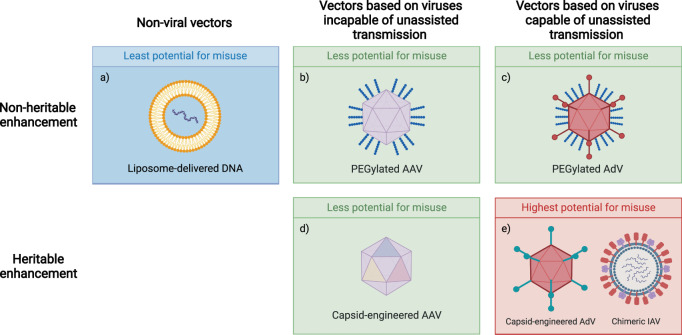
Vector enhancement approaches and associated potential for misuse. **a** Non-viral vector delivery approaches feature the least dual-use potential compared to approaches that involve the modification of viral vectors. **b**, **c** Compared to heritable viral vector enhancement, non-heritable enhancement approaches, such as PEGylation, feature less potential for misuse as they are not passed onto viral progeny. **d** Heritable approaches for enhancement of vectors based on viruses incapable of unassisted transmission, such as AAV, feature less potential for misuse than similar approaches applied to vectors based on viruses capable of unassisted transmission. **e** Compared to other approaches for the enhancement of viral vectors, highest potential for misuse features heritable enhancement of vectors based on viruses capable of unassisted transmission, such as capsid-engineered AdV or chimeric IAV. AAV adeno-associated virus, AdV adenovirus, IAV influenza a virus.

Vectors such as AAV and adenovirus serotype 5 (Ad5) are limited by widespread pre-existing neutralizing antibodies [10], a drawback that can be mitigated through surface protein engineering. For instance, hypervariable regions of the Ad5 hexon protein have been replaced with those of less seroprevalent Ad48 [11]. The same approach could be leveraged to create pathogens able to evade natural or vaccine-induced immunity. Fortunately, the engineering of surface proteins is relatively virus-specific. Since AAV replication requires co-infection with a helper virus, insights specific to that virus are difficult to misuse (Fig. 1d) [12]. However, similar work on vectors whose natural forms are capable of direct and unassisted human-to-human transmission could lead to the generation of novel pandemic agents (Fig. 1e). Particularly concerning insights may arise from capsid engineering or envelope glycoprotein engineering of viruses explored as vectors for oncolytic cancer therapy, such as poliovirus, influenza virus, measles virus, and herpesvirus [13–16].

## Risks from advancing general-purpose viral engineering methodologies

While insights specific to AAV feature little dual-use potential, more accessible and powerful methodologies inspired by its successes may be applicable to viruses with pandemic potential. General-purpose viral engineering methodologies that facilitate higher-throughput or more precise experiments, or that reduce or ameliorate the need for such experiments, could significantly lower the barrier to the generation of novel pandemic agents.

For example, modular and efficient directed evolution methods such as the use of barcoding to achieve high-throughput multiplexed phenotypic analysis of AAV capsids can select for useful viral vector properties, including immune evasion [17]. Similarly, structure-guided rational design applies expert judgment based on cryo-electron microscopy or X-ray crystallography experimental data to achieve a desired outcome [18]. Both involve laborious experimentation and require greater expertise than classical passaging, and consequently feature a high barrier to misuse. Researchers working with such methods share a responsibility to avoid conducting or sharing the results of experiments relevant to potential pandemic pathogens, as many more individuals can assemble agents from genomic blueprints than possess these engineering skills.

Foreseeable future advances could greatly reduce or even obviate the laboratory expertise and equipment currently required for pathogen enhancement. The use of computational viral vector engineering in AAV capsid design was pioneered in 2019 when Ogden et al. used data from a multiplexed phenotypic analysis of an AAV capsid library to train a model for optimizing multiple properties including virus production, immune evasion, thermostability, and biodistribution [17]. Since this initial demonstration, multiple groups have moved into this space [19, 20]. Computational approaches trained on experimental data have since been applied to predict critical loci for AAV capsid assembly and generated functional sequences that are substantially diversified from natural homologs [19, 21]; those drawing on publicly available data on AAV evolutionary and structural features have similarly identified novel viable capsids [20, 22].

While the flexibility and accessibility of computational engineering approaches is highly promising for the therapeutic development of AAVs, these papers highlight their potential applicability to other proteins and viral engineering challenges [17], potentially allowing wet lab scientists lacking expertise in directed evolution and structure-guided rational design to generate novel pandemic pathogens [22]. These risks are exacerbated by protein folding prediction tools, which could generate public training datasets relevant to potential pandemic pathogens, including enveloped viruses [23, 24]. Such computational methods could eventually permit individuals lacking any laboratory expertise beyond the minimum required to generate infectious viruses from synthetic DNA to generate novel pandemic pathogens. Current approaches may still be limited in how easily they may be applied to different viruses, but this is likely to change if researchers continue to explicitly seek to develop flexible and generalizable methods.

### Safer approaches for the advancement of gene therapy

The easiest way to improve vector effectiveness for clinical gene therapy while minimizing dual-use risks is to prioritize methods that are not genetically encodable. For instance, transient pharmacological immune modulation may represent a practical and low-risk alternative to vector engineering for immune modulation. Strategies explored to date include the administration of antibodies against CTLA-4 or pro-inflammatory cytokines, the use of small-molecule proteasome inhibitors, or peripheral induction of immune tolerance to capsids and transgenes with rapamycin [25–27]. Non-genetic modifications of capsids and envelope glycoproteins – for instance, the shielding of surface antigens through PEGylation or with other synthetic polymers [28] or the use of lipid bilayer envelopes to shield vectors from antibody neutralization [29]—are not passed onto viral progeny and may hence represent a low dual-use alternative to capsid engineering (Fig. 1b, c). The use of unnatural amino acids in surface protein engineering, which can open the door to surface modification techniques such as click chemistry, may offer another path to non-heritable immune evasion. Certain viral vectors may also feature properties that remove the need for potentially dual-use viral engineering. For instance, commensal anelloviruses persistently evade the host immune system and may hence not require modifications to overcome anti-vector immunity [30].

Transitioning to non-viral delivery methods would dramatically reduce the potential for gene therapy research to inform the engineering of pandemic pathogens. The development of non-viral delivery methods does not directly generate or spread new capabilities for viral engineering, and may hence feature the least dual-use potential compared to viral vector approaches (Fig. 1a) [31]. One prominent approach for non-viral delivery is the use of lipid nanoparticles (LNPs) as a flexible delivery system for siRNA, mRNA, DNA, or gene editing complexes [32]. While non-viral vector delivery methods have previously been limited by poor delivery efficiency, non-viral vectors are increasingly being investigated in clinical trials [33]. Non-viral delivery methods feature advantageous properties such as ease of manufacturing, reduced immunotoxicity, larger payloads, and flexibility of design [32]. Importantly, non-viral delivery methods do not induce neutralizing anti-vector antibodies and may be suitable for repeat administrations [34]. However, non-viral delivery systems still face numerous challenges, including targeting specific sites and cell types; advances in lipidomics may overcome this limitation [35]. Preferential investigation of non-viral delivery methods may constitute an effective strategy to advance gene therapy while robustly mitigating dual-use risks.

### Mitigating risks from the advancement of computational protein engineering

The application of computational protein engineering to virus entry, not just capsid or envelope glycoprotein immune evasion, could also be used to generate or enhance pathogens with pandemic potential. To safeguard the transformative potential of this technology and protect human health [36], developers of computational tools for biological sequence design should consider how to mitigate the associated risk for misuse.

Enhanced AAV is not concerning in itself as it requires a helper virus for human-to-human transmission. However, the tools developed for the engineering of AAV feature dual-use potential if applied to other viruses. A first important step should be to limit the development and application of models for viral engineering to the least concerning agents, including AAV and other viruses incapable of unassisted human-to-human transmission, until the establishment of a mechanism capable of ensuring that code and training datasets are only accessible for legitimate research purposes. This is critical because sharing code for the modification of viral properties of viral families with pandemic potential irreversibly lowers the barrier to the enhancement of the respective pathogen. Deliberations on a strategy for non-physical viral engineering tools that balances accessibility and mitigation of misuse should be initiated by the U.S. Office of Science and Technology Policy in conjunction with the National Security Council, and equivalents in other nations, to avoid the conflicts of interest faced by health agencies. In conjunction, open-source platforms such as GitHub and Open Science Framework should proactively champion biosecurity by restricting access to general-purpose code that could plausibly be used to generate or enhance potential pandemic pathogens.

### Take-aways for the governance of dual-use research

Our findings underline the importance of mitigating biosecurity risks in fields such as gene therapy, vaccinology, and oncolytic virus research, which have not traditionally been associated with such concerns. While some countries, including the United States, have nascent policies to regulate dual-use risks, such frameworks currently don’t capture risks from insights transferable between agents, nor from technologies outside of biology, and are typically implemented by agencies lacking security expertise that directly sponsor the relevant research [37, 38]. To address this deficit, science agencies without such conflicts of interest should update policies to cover the development of viral engineering insights and approaches developed for less concerning agents which are readily transferable to pathogens with pandemic potential. Deliberations on this topic should involve the private sector given its importance in advancing cutting-edge technologies. Given the transnational nature of modern biomedical sciences research, respected international scientific authorities such as the World Health Organisation and the InterAcademy Partnership should develop global norms for dual-use research oversight.

## Conclusion

Gene therapy holds enormous promise for human health. However, some lines of viral vector research may make it substantially easier to generate or enhance pandemic pathogens. To minimize this risk, we recommend preferentially advancing lines of research with less potential for misuse. When methods with dual-use potential are pursued, effective solutions to mitigate misuse must be created. In a time of increasing biotechnological capabilities accessible to a growing number of individuals, proactively mitigating the potential for well-meaning research to be misused will protect human health and safeguard future biomedical advances.
